# Testosterone profile in older men with Alzheimer’s disease

**DOI:** 10.1590/S1980-57642009DN20400010

**Published:** 2008

**Authors:** Cristiana Roscito Arenella Dusi, Lílian Schafirovits Morillo, Regina Miksian Magaldi, Adriana Nunes Machado, Sami Liberman, Wilson Jacob Filho

**Affiliations:** Department of Geriatric, School of Medicine of University of São Paulo, Brazil

**Keywords:** testosterone, cognition, Alzheimer’s disease

## Abstract

Evidence suggests low testosterone levels in Alzheimer’s disease.

**Objectives:**

To compare testosterone levels between older men with and without Alzheimer’s disease.

**Methods:**

Fourteen men with Alzheimer’s disease were compared with twenty eight men without dementia. Demographic variables and clinical profiles were analyzed. Within fifteen days before or after the described evaluation, measures of total testosterone and Sex Hormone Binding Globulin (SHBG) were performed. Free testosterone level was calculated based on total testosterone and SHBG. Quantitative variables were analyzed using Student’s t test or Kruskal-Wallis test, while qualitative variables were analyzed using chi-square or Fisher test.

**Results:**

Mean age in the Control and Alzheimer’s disease groups were 72.0 (SD±4.8) years and 79.3(SD±5.9) years, respectively (p=0.001). Mean schooling between these two groups were 8.78 and (±5.86) years, respectively (p=0.022). There were no statistically significant differences between the two groups for testosterone levels, although a trend was observed for the Alzheimer’s disease group to present lower levels than the control group (p=0.066). There was no direct correlation between free testosterone and age, although a trend was evident (p=0.068).

**Conclusions:**

There was no significant difference in testosterone between men with AD and those without dementia.

## Abstract (pt)

Evidências sugerem testosterona reduzida na doença de Alzheimer (DA).

**Objetivos:**

comparar níveis de testosterona entre homens idosos com doença de Alzheimer e sem demência.

**Métodos:**

Comparamos 14 indivíduos com doença de Alzheimer leve e moderada, provável e possível e 28 sem demência. Analisamos variáveis demográficas e perfil clínico. Em um período de quinze dias antes ou depois da avaliação clínica, foram realizadas coletas séricas de testosterona total e SHBG. Testosterona livre foi calculada baseada na testosterona total e SHBG. Variáveis quantitativas foram analisadas pelo teste T-Student (paramétricas) ou Kruskal-Wallis (não-paramétricas); variáveis qualitativas, pelo teste Qui-quadrado ou de Fisher.

**Resultados:**

Médias de idade nos grupos Controle e DA foram 72,0 (±4,8) e 79,3 (±5,9) anos, respectivamente (p=0,001). Médias de escolaridade entre os grupos foram de 8.78 e 5.86 anos, respectivamente (p=0,022). Não houve diferença estatisticamente significativa entre os níveis de testosterona livre nos dois grupos, embora se observasse uma tendência do grupo DA a apresentar níveis menores que o controle (p=0,066). Não houve correlação direta entre testosterona livre e idade, embora seja possível observar uma tendência (p=0,068).

**Conclusões:**

não houve diferença significativa quanto aos níveis de testosterona entre indivíduos com DA e sem demência.

Population aging is accompanied by an increase in cases of dementia, particularly Alzheimer’s disease (AD). The prevalence of dementia doubles every 5 years after 65 years of age, reaching 15 to 20% after the 75th year.^1^ There are many studies have been conducted to unravel the pathological mechanisms, risk factors, protective factors and pharmacological agents able to halt or modify the course of AD. Currently, one of the aspects under study is the correlation between cognitive disorders and sex hormones in the elderly.

The aging process is accompanied by a spontaneous decline in the production of sex hormones. In elderly men the production of testosterone gradually falls.^2,3^ Many older men have testosterone levels at the lower limit of normal. The longitudinal study of Baltimore,^4^ developed by the National Institute on Aging (NIA – National Institute on Aging, USA), showed a prevalence of low levels of total testosterone in elderly men aged over 60 (19%), 70 (28%) and 81 years (49%) and low levels of free testosterone index (FTI=testosterone/SHBG) in men aged over 60 (34%), 70 (68%) and 80 years (91%) in the descriptive assessment of the original sample. This study has given rise to numerous important studies cited below.

The most important androgen is testosterone. The receptors of androgen and estrogen are found in high concentrations in male sexual organs and in some other areas of the body including the brain, skeletal muscle, heart, vascular smooth muscle and bone. The areas of the brain most involved are hippocampus and amygdala, responsible for memory and learning.^5^ The concentrations of the androgen receptor in different tissues is influenced by age with reports of decreased levels with aging.^3^

Circulating testosterone is predominantly (50-60%) through a strong link with the protein carriers of sex hormones (SHBG). Approximately 1 to 2% of circulating testosterone is free and 40 to 50% is weakly bound to albumin.^3^ The concentrations of free and albumin-bound testosterone represent the testosterone biologically available to the body.

The testosterone seems to develop a neuroprotective role in the development of Alzheimer’s disease. Studies suggest that testosterone inhibits hyperphosphorylation of tau protein and formation of amyloid-β protein.^6^ Other possible mechanisms of action regarding the neuroprotection include increased concentration of nerve growth factor (NGF) and the anti-apoptotic^6^ effect.

Moffat et al.^7^ conducted longitudinal study in Baltimore where 574 individuals were assessed for an average period of 19.1 years. This prospective study investigated the relationship between reduced levels of total testosterone and free testosterone index associated with age and subsequent development of Alzheimer’s disease. The individuals were subjected to physical, neurological and neuropsychological evaluations every two years. The hormone measurements occurred at the same time as the ratings. It was concluded that the levels of free testosterone at initial assessment and at last hormone measurement showed an association with AD. The results showed that for every 10-unit (nmol/nmol) increase in free testosterone index the risk of developing AD falls by 26%.

The relationship between sex hormones and cognition, however, remains uncertain, where some studies have shown no correlation, such the investigation by Pennanen et al.^8^ which demonstrated high levels of testosterone in AD individuals.

## Objectives

To compare levels of testosterone among individuals with mild or moderate Alzheimer’s disease and individuals without dementia.

## Methods

A total of 41 men aged more than sixty years with AD were selected from the Reference Center for Cognitive Disorders (CEREDIC) and the Department of Neurology, both part of the Hospital of the University Medical School of Sao Paulo. Fourteen patients agreed to participate in this study. Four patients did not participate in accordance with the criteria for exclusion, 6 had difficulty in transportation, 4 had no physical and/or behavioral condition, 1 family was not available to collaborate, 2 patients were traveling, 1 patient was institutionalized and 9 families did not allow the patient to participate. All the patients in question had a diagnosis of AD according to the criteria of the Diagnostic and Statistical Manual of Mental Disorders, Fourth Edition (DSM-IV). This study includes patients with probable and possible AD, according to the NINCDS-ADRDA criteria (Working Group of the National Institute of Neurology and the Association of Alzheimer’s disease and Related Disorders USA).

There were 31 men aged more than sixty years, without dementia, accompanied by the Multidisciplinary Assistance Clinic for the Aged (GAMIA) under the Geriatrics Division of the Clinicas Hospital of University of São Paulo. Twenty-eight elderly agreed to take part in this work and only 3 people did not accept.

The groups were grouped according to criteria of the Clinical Dementia Rating^9^ (CDR) into Controls (Group I) formed by individuals without dementia (CDR-0), or patients (Group 2) with mild (CDR-1) or moderate AD (CDR-2).

### Exclusion criteria

#### AD–CDR 3

- Depression with symptoms, or patients who had their antidepressant medication changed six months or less before the trial;
- Potentially reversible dementias;
- Hormone replacement therapy with testosterone;
- Use of drugs that hamper interpretation of plasma levels of testosterone: glucocorticoids, analogues of gonadotropin-releasing hormone, antiandrogens, blocking 5-alpha-reductase, chemotherapy with alkylating, Ketoconazole, Spironolactone, opioids.

### Socio-demographic profile

The socio-demographic variables analyzed were age, education, marital status, occupation, religion and clinical profile of individuals: smoking, drinking, body mass index (BMI), history of cancer, coronary artery disease, diabetes mellitus, hypertension, heart failure, stroke, hyperthyroidism, hypothyroidism, benign prostatic hyperplasia, osteoporosis, auditory and visual acuity complaints and current medications.

The study was approved by the Ethics Committee of the Medical Hospital of the Faculty of Medicine of the University of Sao Paulo. All individuals and/or their caregivers signed a free and informed consent term.

At the end of this study all subjects had received a letter at their residences containing their cognitive performance and results of hormone examinations. Individuals were suggested to send their results to their personal doctor.

### Hormonal measures

Blood sample were collected within a period of fifteen days before or after the clinical evaluation, always in the morning, because of circadian fluctuations of testosterone. The subjects underwent fasting for four hours. The examinations were conducted at the Central Laboratory of the Hospital of the Faculty of Medicine of the University of Sao Paulo.

Serum levels of total testosterone were evaluated by the Fluoroimmunoassay method (AutoDELFIA Wallace, Turku, Finland) while levels of SHBG were ascertained using the method immunofluorometric method (AutoDELFIA Wallace, Turku, Finland). The result of free testosterone was based on the calculation of total testosterone and SHBG described by Vermeulen.

TT (ng/dl)/28/84=TTnmol/L

X=(SHBG)–(TT)+23.32

Y=(TT)×93.28

FT(pmol/L)=[–X+square root (X^2^ +Y/46.64)]×1000

Where: TT=total testosterone, FT=free testosterone.

### Statistical analysis

Univariate and descriptive analysis was performed for both groups. Quantitative variables were analyzed by Student’s t test or the non-parametric Kruskal-Wallis test. Qualitative variables were compared using the chi-square or Fisher exact test.

## Results

The mean age of the control group was significantly lower than the AD group (72.0±4.8 years versus 79.3±5.9 years, p=0.001) and the average educational level was significantly higher (8.78±4.7 years versus 5.86±4.1 years (p=0.022).

The clinical characteristics of the study population are shown in Table 1.

**Table 1:** Clinical profile of control and Alzheimer's groups.

Variables	Control ( I ) n=28	Alzheimer's disease (II)n=14
Smoking	0	2 (14.2%)
Former smoking	18 (64.2%)	9 (64.2%)
Alcohol	14 (50%)	2 (14.2%)
Former alcohol	3 (10.7%)	4 (28.5%)
BMI		
<21.9	3 (10.7%)	3 (21.4%)
22 to 27	14 (50%)	9 (64.2%)
27.1 to 30	8 (28.5%)	2 (14.2%)
>30.1	3 (10.7%)	0
Hypertension	20 (71.42%)	9 (64.2%)
Diabetes	2 (7.14%)	3 (21.4%)
Stroke	2 (7.14%)	2 (14.2%)
CHD	8 (28.5%)	5 (35.7%)
Dyslipidemia	14 (50%)	10 (71.4%)
Heart Failure	3 (10.7%)	1 (7.14%)
Cancer	2 (7.14%)	0
Hyper/ hypothyroidism	2 (7.14%)	1 (7.14%)
Osteoporosis	1 (3.5%)	0
HPB	3 (10.7%)	2 (14.2%)
Depression	1 (3.5%)	6 (42.8%)
Visual impairment	1 (3.5%)	1 (7.14%)
Hearing impairment	9 (32.1%)	5 (35.7%)

BMI, body mass index; CHD, coronary heart disease; HPB, benign prostatic disease.

The AD group tended to have lower levels of average free testosterone (222.4 pmol/L, ±101.8) than the control group (272.3 pmol/L, ±96.1) reaching a borderline statistical difference (p=0.06), and tended to have higher average levels of SHBG 73.1 (SD±32.7) and 56.2 nmol/L (SD±26.8). The results did not reach statistical significance (p=0.072).

The average total testosterone between the groups were similar, as indicated in Table 2.

**Table 2:** Hormonal measures between control and Alzheimer's groups.

Hormonal measures		Groups		
		Alzheimer's disease	Control	p
Total testosterone ng/dL	Minimum	295	214	-
	Maxim	710	897	-
	Mean (SD)	461.9 (123.3)	4813 (196.1)	p=0.98
Free testosterone pmol/L	Minimum	105	133	-
	Maxim	505	521	-
	Mean (SD)	222.4 (101.1)	272.3 (96.1)	p=0.06
SHBG nmol/L	Minimum	31	25	-
	Maxim	146	142	-
	Mean(SD)	73.1 (32.8)	56.2 (26.9)	p=0.07

SD, standard deviation.

The correlation between age and free testosterone was not statistically significant (p=0.068), but suggested that the higher the age, the lower the serum level of free testosterone (Table 3).

**Table 3:** Comparison of age and education between control and AD groups.

Variables		Control group (n=28)	AD group (n=14)	Statistical analysis
Age	Minimum	62	71	-
	Maxim	86	89	-
	Mean(SD)	72 (4.8)	79.3 (5.9)	0.001
Education	Minimum	3	3	-
	Maxim	17	15	-
	Mean(SD)	8.78 (4.7)	5.86 (4.1)	0.022

SD, standard deviation.

## Discussion

The results of this cross-sectional study showed no difference in levels of testosterone among patients with Alzheimer’s disease (mild or moderate) and the group without dementia. The AD group presented a trend toward lower levels of free testosterone and higher levels of SHBG.

The production of total and free testosterone declines gradually with age. Since the AD group was older than the control group, it is possible that the observed trend of correlation between testosterone and AD was mediated by the age factor. Therefore, the assessment of levels of testosterone in the groups suggests that the variable was an age bias. Lopes et al^10^ found a positive correlation between hearing impairment and mild cognitive impairment in elderly patients through a cross case-control study. The group of patients with cognitive disorders was older than the control group and this difference was statistically significant where age was also found to be a bias factor in the cited study. Thus, many trials have encountered the same difficulty in assessing elderly people with cognitive disorder, who are usually very old.

Few studies have investigated the relationship between testosterone and neurodegenerative diseases such as Alzheimer’s disease while results to date have been contradictory. Hogervorst et al.^11^ found similar results to the present study. His group cross developed a case-control study which evaluated 83 patients with AD and 103 patients without cognitive disorder. The mean age was 75 years (SD±9) with no statistical difference between the groups. This study showed low levels of total testosterone in elderly men with Alzheimer’s disease. In contrast, among women there was no difference in levels of total testosterone between AD and controls. Suravarapu et al.^12^ showed a higher incidence of dementia in patients with lower levels of total and bioavailable testosterone, although the results were not statistically significant. Perhaps the main limitation of this study was that 36.7% of the original sample was composed of young people between 60–69 years. The short length of follow-up was another limiting factor leading to a small number of diagnosed dementia cases.

Moreover, Pennanen et al.^8^ performed a cross-sectional study comparing the testosterone levels in fourteen men with AD and sixteen men without dementia. There was no statistical difference in the age variable between the groups. This study concluded that patients with AD showed high levels of total testosterone, free testosterone index and free androgen (free androgen index). No differences were found with respect to levels of SHBG.

Because of the scarcity of studies in the medical literature on this topic it was difficult to deepen the discussion, highlighting the need for further studies that address the correlations between hormones and cognition.

One of the limitations of this study was in evaluating testosterone not linked to albumin. It seems that biological function introduces bioavailable testosterone in the body and therefore a fraction of testosterone was not examined in this study that could have acted on cognitive function.

In view of the limitations of this study outlined future studies are warranted that include assessments of bioavailable testosterone and groups which are matched for age.

## References

[1] Freitas EV, Py L, Cançado FAX, Doll J, Gorzoni ML (2006). Tratado de Geriatria e Gerontologia.

[2] Morley JE, Perry HM (2003). Andropause: an old concept in new clothing. Clin Geriatr Med.

[3] Kaufman JM, Vermeulen A (2005). The decline of androgen levels in elderly men and its clinical
and therapeutic implications. Endocr Rev.

[4] Harman SM, Metter EJ, Tobin JD (2001). Longitudinal effects of aging on serum total and free
testosterone levels in healthy men. J Clin Endocrinol Metab.

[5] Beauchet O (2006). Testosterone and cognitive function: current clinical evidence of
a relationship. Eur J Endocrinol.

[6] Bialek M, Zaremba P, Borowicz KK (2004). Neuroprotective role of testosterone in the nervous
system. Pol J Pharmacol.

[7] Moffat SD, Zonderman AB, Metter EJ (2004). Free testosterone and risk for Alzheimer disease in older
men. Neurology.

[8] Pennanen C, Laakso MP, Kivipelto M (2004). Serum testosterone in males with Alzheimer´s
disease. J Neuroendocrinol.

[9] Morris JC (1993). The clinical dementia rating (CDR): current version and scoring
rules. Neurology.

[10] Costa LL, Magaldi RM, Gândara MER (2007). Prevalence of hearing impairment in patients with mild cognitive
impairment. Dement Neuropsiychol.

[11] Hogervorst E, Williams J, Budge M (2001). Serum total testosterone is lower in men with Alzheimer's
disease. Neuro Endocrinol Lett.

[12] Suravarapu SMD, Bergstralh EJ, Farmer A (2006). Dementia and low testosterone and bioavailable testosterone
levels in men. Alzheimer Dis Assoc Disord.

